# A Novel Approach for Localizing Non‐Sustained Atrial Arrhythmias: Atrial Pace‐Mapping With Automatic Intracardiac Pattern Matching

**DOI:** 10.1111/jce.16734

**Published:** 2025-06-03

**Authors:** Evgeny Lian, Vera Maslova, Sven Willert, Adrian Zaman, Derk Frank, Fabian Moser

**Affiliations:** ^1^ Department of Internal Medicine III (Cardiology and Intensive Care Medicine) University Hospital Schleswig‐Holstein (UKSH) Kiel Germany; ^2^ German Centre for Cardiovascular Research (DZHK), partner site North Kiel Germany

**Keywords:** atrial fibrillation, atrial tachycardia, automatic technique, catheter ablation, non‐pulmonary vein trigger, pace mapping

## Abstract

**Introduction:**

Ventricular pace mapping is an established tool to identify the origin of non‐inducible arrhythmias by analyzing the paced QRS morphology of the surface electrocardiogram (ECG). Using the same approach for atrial pace mapping (APM) was shown to be suboptimal, as accurate assessment of P wave morphology can be limited. We present a novel approach for APM using an automatic ECG pattern‐matching algorithm with intracardiac unipolar signals (aICPM).

**Methods and Results:**

Forty‐five consecutive patients presenting with non‐sustained atrial tachycardia (nsAT) or non‐pulmonary vein (PV) triggers were prospectively included. APM using aICPM was performed with six biatrial unipolar signals to create score maps. Ablation targeted sites with the best intracardiac pattern similarity. The primary endpoint was defined as successful localization and non‐inducibility of the arrhythmia. Secondary endpoint was defined as freedom from AF/AT during the follow‐up.

APM with aICPM successfully identified specific areas with high intracardiac pattern similarity in all patients. The median time required to create a score map was 5.0 (IQR 3.3; 6.3) minutes, with 106 (IQR 77; 155) points per map. Radiofrequency ablation was performed successfully in all but two patients, with a median ablation time of 134 (IQR 75; 180) seconds and an ablation area of 2.0 (IQR 1.1; 2.3) cm². Two patients underwent ethanol ablation of the vein of Marshall. All cases achieved non‐inducibility of the arrhythmia. During a follow‐up of 5.9 ± 1.87 months, five patients experienced arrhythmia recurrence.

**Conclusion:**

This novel approach rapidly and accurately identifies the origin of atrial arrhythmias by creating atrial pacemaps using an automated ECG pattern‐matching algorithm, which processes intracardiac unipolar signals.

**Abbreviations**
AF: atrial fibrillation
aICPM: automatic intracardiac pace mapping
APM: atrial pace mapping
AT: atrial tachycardia
CS: coronary sinus
HRA: high right atrium
IC: intracardiac
nsAT: non‐sustained atrial tachycardia
PAC: premature atrial contraction

## 1 Introduction

Identifying the origin of focal atrial arrhythmias can be challenging [1]. However, it becomes even more challenging, if the ectopic focus is not sustained during the course of the procedure [2]. Ablation of non‐PV triggers initiating AF has been shown to improve outcomes [3]. The transient and unstable character of these triggers makes the exact localization oftentimes difficult [3, 4, 5]. Precise activation mapping of the focal source requires ongoing tachycardia. Often, only a single premature atrial contraction (PAC) or atrial tachycardia (AT) run immediately triggers non‐mappable AF, with limited use of conventional activation mapping [6]. Atrial pace mapping (APM) is a method used to localize unstable focal arrhythmias. It relies on comparing P‐wave patterns on surface electrocardiogram (ECG). However, the low signal‐to‐noise ratio and overlap with T‐waves limit its clinical use [7]. Analysis of P‐wave morphology offers only limited information for localizing the exact site of focal atrial arrhythmias, especially in patients with diseased atria [8]. APM incorporating intracardiac (IC) signals from stable referent electrodes improves its accuracy but still relies on subjective visual analysis of IC signals [9]. Yamashita et al. recently assessed the feasibility of APM with an automatic intracardiac pace mapping (aICPM) algorithm for ablation of unstable ATs and PACs in 22 patients. Using unipolar signals from coronary sinus (CS) and RA referent catheters IC pattern similarity was calculated. Despite its advantages, this approach has limitations, including manual assignment of matching values to activation times and the time‐consuming acquisition of low‐density maps (9–12 points per map). Nonetheless, aICPM adds objectivity and simplicity to APM [10].

The EnsiteX system (Abbott Laboratories, MA, USA) lacks a dedicated aICPM algorithm but offers an ECG pattern‐matching tool for the automatic creation of the pattern similarity score maps without requiring manual reassignment, enabling faster, more efficient mapping [2]. In this study, we assessed the feasibility of a novel workflow for APM using aICPM with IC unipolar signals and the ECG matching algorithm.

## 2 Methods

The study was conducted in three phases: (1) Definition of similarity score cutoff for the IC patterns from the same origin; (2) Validation of the reliability and accuracy of APM using aICPM in sustained rhythms; (3) Assessment of APM feasibility using aICPM for localizing non‐PV triggers.

**Figure 1:**
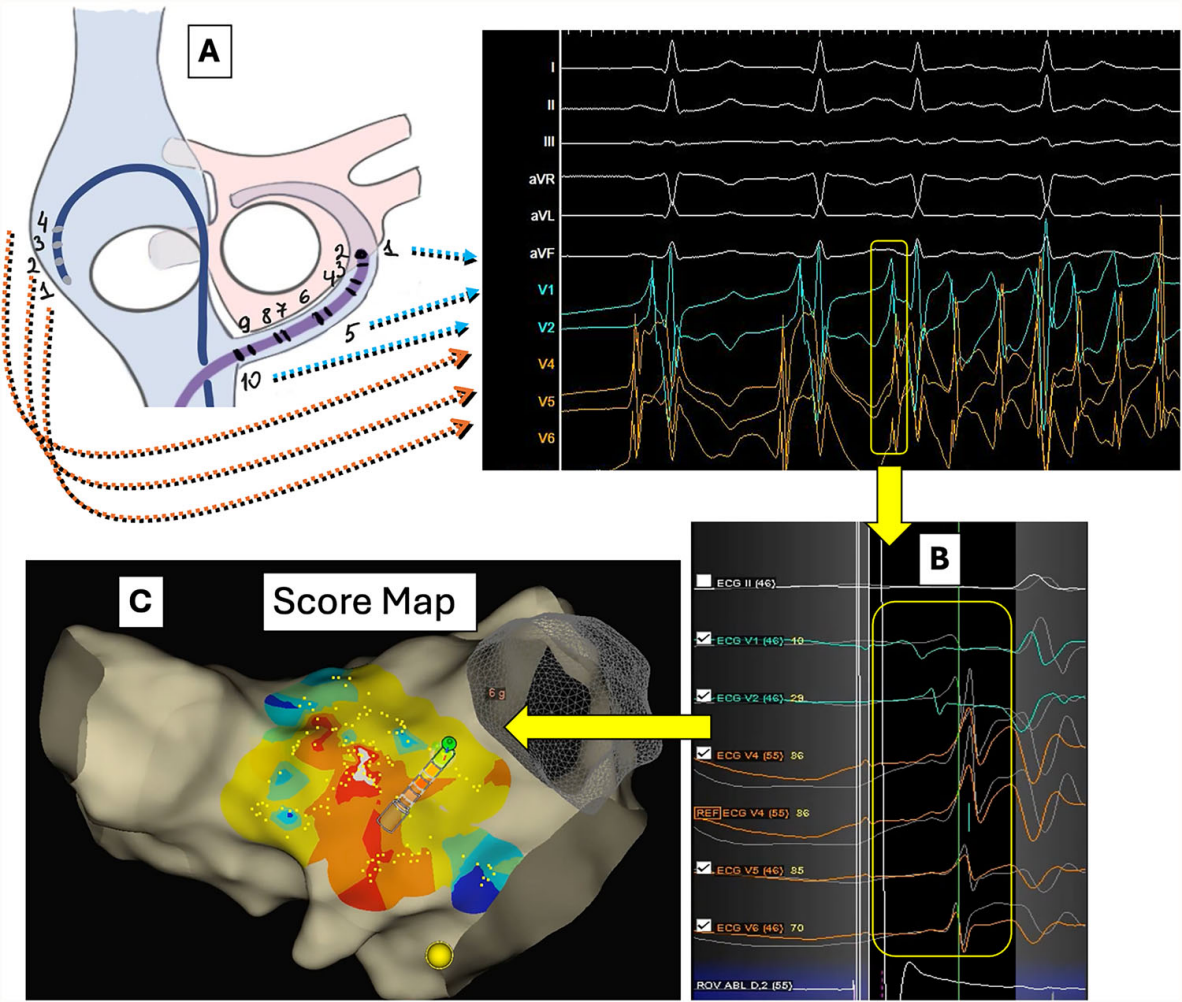
The concept of atrial pace mapping with automatic intracardiac pattern matching using an ECG matching algorithm. (A) Biatrial unipolar signals passed to the precordial leads via a custom‐made adapter. (B) IC pattern of the AF‐trigger is acquired as a template for pace mapping. (C) A score map created by APM with aICPM using an ECG pattern matching algorithm fed by biatrial unipolar signals. AF, atrial fibrillation; aICPM, automated intracardiac pattern matching; APM, atrial pace mapping; IC, intracardiac; SR, sinus rhythm.

**Figure 2:**
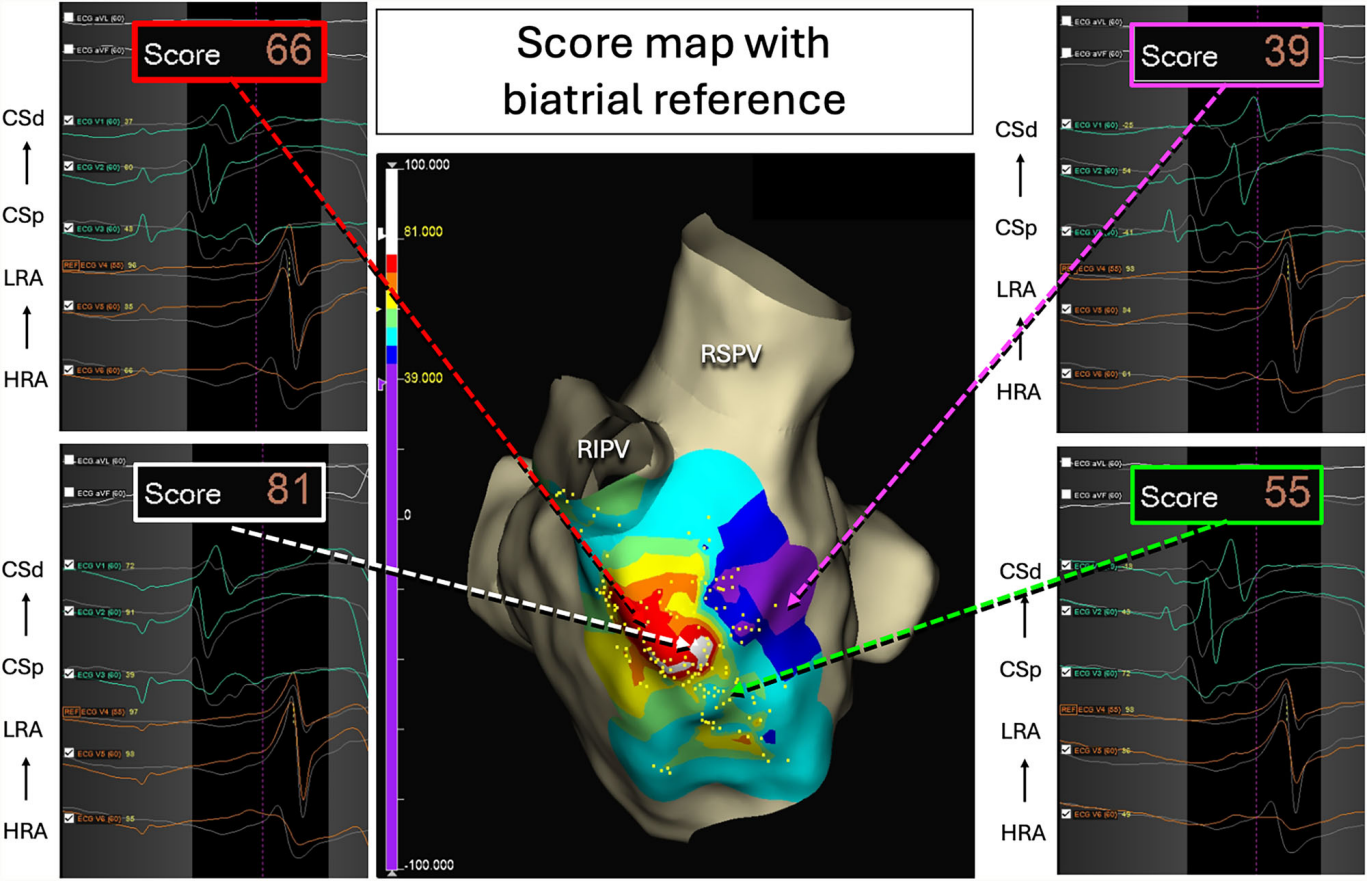
A score map of non‐PV trigger in the left atrium created by atrial pace mapping with biatrial reference and examples of different degrees of intracardiac pattern matching. CSd, coronary sinus distal; CSp, coronary sinus proximal; HRA, high right atrium; LRA, low right atrium.

Study population and procedure setup: Consecutive patients who underwent an ablation procedure for sustained AT or first‐do AF (Phases 1 and 2) or AF recurrence with induction of non‐PV triggers (Phase 3) using the EnsiteX system between January and July 2024 were prospectively enrolled. Patients with detected low‐voltage areas underwent substrate modification without non‐PV trigger mapping and were excluded from the study. Procedures were performed under conscious sedation. A deflectable decapolar catheter was placed in the coronary sinus (CS), and a non‐deflectable quadripolar catheter in the high right atrium (HRA). Three electrodes from CS and three electrodes from the HRA catheter were connected to the precordial leads via a custom adapter to pass the unipolar signals to the ECG pattern matching algorithm as described previously (Figure 1, Supporting Information S1: Figure 1) [8]. The standard filters were applied for the noise cancellation and reduction of respiration effects (0.5 Hz; 50 Hz; notch filter On). Additional respiration compensation algorithm ensured the accurate location of the acquired points on the score map. An IC pattern template was defined by setting the the matching window between the first and last inflexion points of the unipolar signals. The ECG matching algorithm calculated scores based on waveform correlations within this window for each lead (value range −100 to +100%, Figure 2). The total similarity score averaged the matching scores for the V1‐V6 leads. Fluoroscopic control, impedance‐based catheter visualization in the 3D navigation system and the IC pattern of sinus rhythm (SR) were used to track the position stability of the referent electrodes (Central illustration 1, 3).

**Central illustration 1:**
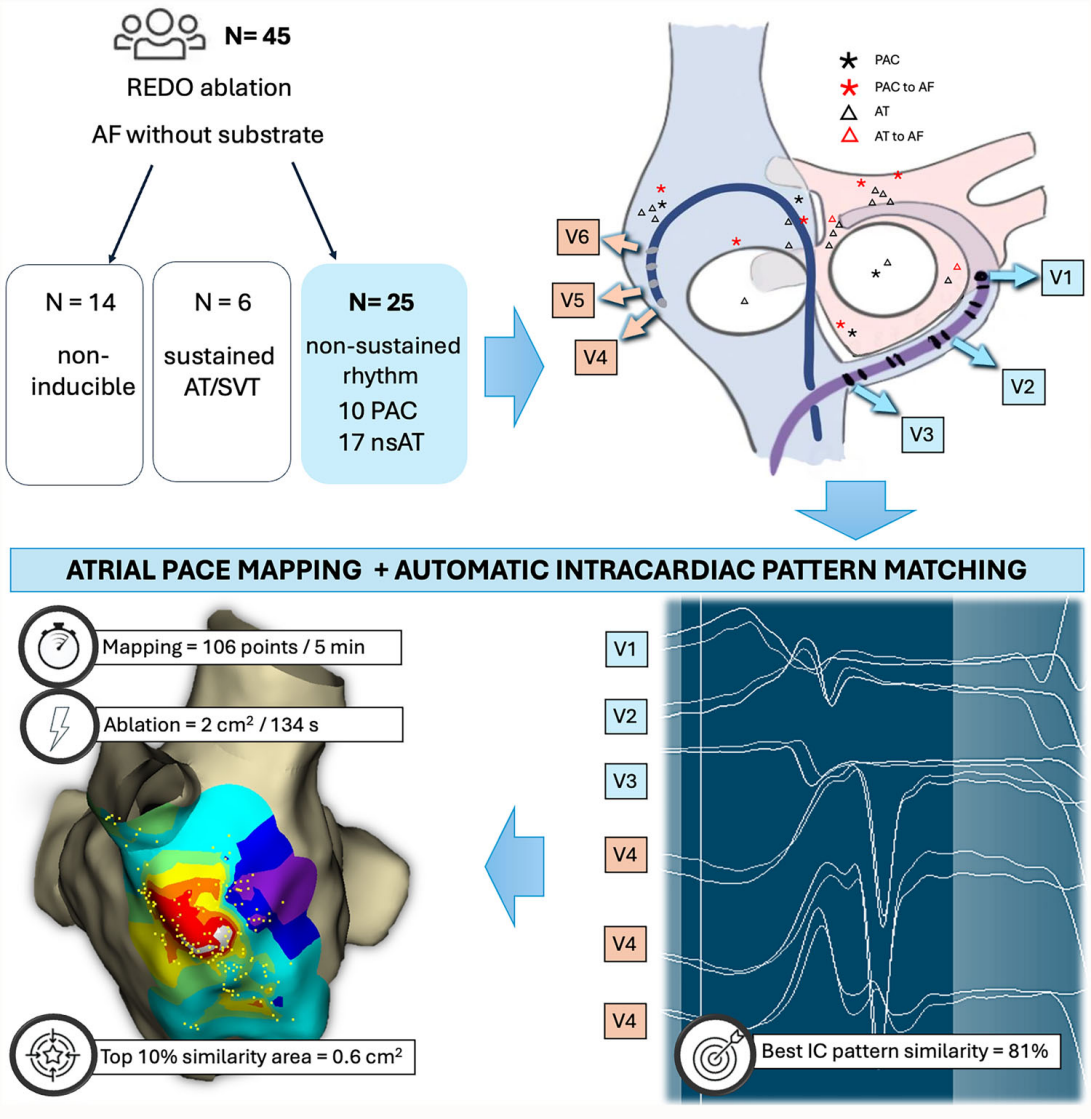
The flowchart of the study and principles of the atrial pace mapping using biatrial intracardiac unipolar signals, passed to the automatic ECG matching algorithm for the rapid generation of a detailed score map. The novel approach precisely localizes the origin of non‐PV triggers to guide individualized catheter ablation.

Phase 1. Definition of similarity score cutoff for the IC patterns from the same origin.

The IC patterns from the same site of origin (SOO) cannot be 100% ideally similar due to the fluctuations in the atrial IC unipolar EGM morphology related to micromovement during breath or heartbeat cycle and overlaying the different phases of the ventricular activation (QRS, ST‐T waves). To define the optimal cutoff value which still ensures the same origin of the rhythm, the two sets of IC pattern score maps were created, and the dispersion similarity score was assessed: one for the stable rhythm from the SOO and one for paced IC patterns outside SOO. For the same SOO score map, an IC pattern of the first atrial beat was acquired and automatically matched to the subsequent IC morphologies without catheter manipulation to exclude the mechanically induced beats. For the score map with the beats outside the SOO, the IC pattern of the SOO was automatically matched to the paced morphologies from different locations outside the SOO. The score maps were exported for the offline analysis of IC pattern similarity dispersion and for calculating the optimal cutoff values for the rhythms of the same SOO.

Phase 2. Accuracy of APM using aICPM of the sustained rhythms

To assess the reliability and accuracy of the method, APM was performed in patients with sustained focal AT or with paced atrial rhythms. The SOO of the rhythm was identified by activation mapping of sustained focal AT or set artificially by pacing with CL 500 ms. The SOO IC pattern was acquired as a template and APM was performed in both atria using a 4 mm tip contact‐force‐enabled ablation catheter (CL of 500 ms, output 5 to 10 V, duration of 1 ms), starting near the earliest IC unipolar signal to exclude the pacing artefact from the matching window. One of the IC unipolar signals was set as a referent for the matching window offset. Matching scores were calculated only using leads V1–V6 (IC unipolar atrial signals), and a score map was created with color‐coded similarity values (0%–100%). The precision of APM was assessed by calculating the area of the top 10% similarity.

Phase 3: Feasibility of APM with aICPM for localizing non‐PV triggers

Consecutive patients who underwent redo procedure for recurrence of AF after initial PVI were prospectively included. After the transseptal puncture, a high‐density mapping was performed, and if the PV reconnection was revealed, it was abolished by segmental RePVI. Only patients with normal voltage detected by electroanatomical mapping were included. Patients with detected low voltage underwent substrate modification and were excluded from the study. If sustained AT or SVT were induced by programmed or burst atrial and ventricular pacing, they were mapped and ablated accordingly. Otherwise, the induction of non‐PV trigger (PAC or nsAT) was performed using the high‐dose isoproterenol infusion (starting at 20 μg/min and up to 30 μg/min). The IC pattern of every PAC or nsAT was stored and saved. To assure the clinical relevance of the PAC or atrial tachycardia, the pattern was used as an IC template for pace mapping, when reproducible beats from a PAC or an atrial tachycardia showed a similar IC pattern—indicating a reproducible focus with the same site of origin. Reproducible induction of the same focus was defined as at least three occasions of the PAC or nsAT with the IC‐pattern similarity above the threshold specified in Phase 1. If PAC or nsAT initiated AF, electrical cardioversion was performed, and the IC pattern of the first beat was used as a template for APM, with no further induction attempts to avoid additional cardioversion.

Atrial pace mapping was performed using a 4 mm tip contact‐force catheter. Atrial pacing output was reduced (5 to 10 V, duration of 1 ms) to assure local capture. The paced cycle length was set according to the recorded nsAT clycle length, the coupling interval of the non‐PV trigger or, when pacing with this cycle length was not feasible, to a fixed cycle length of 500 ms. One reference catheter, recording the unipolar right atrial signals, was positioned at the RA lateral wall and another reference catheter, recording unipolar left atrial signals, was positioned in the mid coronary sinus. Pace‐mapping was usually performed in both atria. However, biatrial unipolar recordings from both reference catheters help to guide to the chamber of interest.

### 2.1 Ablation and Follow‐Up

Ablation (50 W) targeted the site of the best IC pattern similarity, covering the area of the top 10% similarity (Supporting Information Video S1). The induction protocol was repeated to confirm trigger elimination. Patients underwent standard 48‐h Holter monitoring follow‐up. Any atrial arrhythmia episode over 30 s recorded by ECG was defined as a recurrence.

The endpoint for Phase 3 was the successful localization of the non‐PV trigger origin and non‐inducibility after ablation.

### 2.2 Statistical Analysis

Continuous variables were presented as median (interquartile range [IQR]), and the Mann–Whitney *U* test was used to compare them. The *χ* ^2^ test and Fisher exact test were used for the categorical variables. Two statistical methods were used to calculate the optimal IC pattern similarity cutoff for the rhythms of the same origin: (1) inflexion point analysis of the Kernel Density Estimation (KDE) curve for IC pattern similarity distribution based on its derivative, and (2) ROC curve analysis.

This study protocol complied with the Declaration of Helsinki and was approved by the institutional board.

## 3 Results

### 3.1 Phase 1. IC Pattern Similarity Score Cutoff for the Same Origin

IC pattern matching score maps were collected in 17 patients (5 with sustained focal AT, 12 with reentry AT). The median number of points per map was 848 (IQR 460; 1016). The median IC pattern similarity for all ATs was 86% (IQR 75%; 94%) with a median standard deviation of 13 (IQR 7;19) (Supporting Information S1: Table S1).

**Figure 3:**
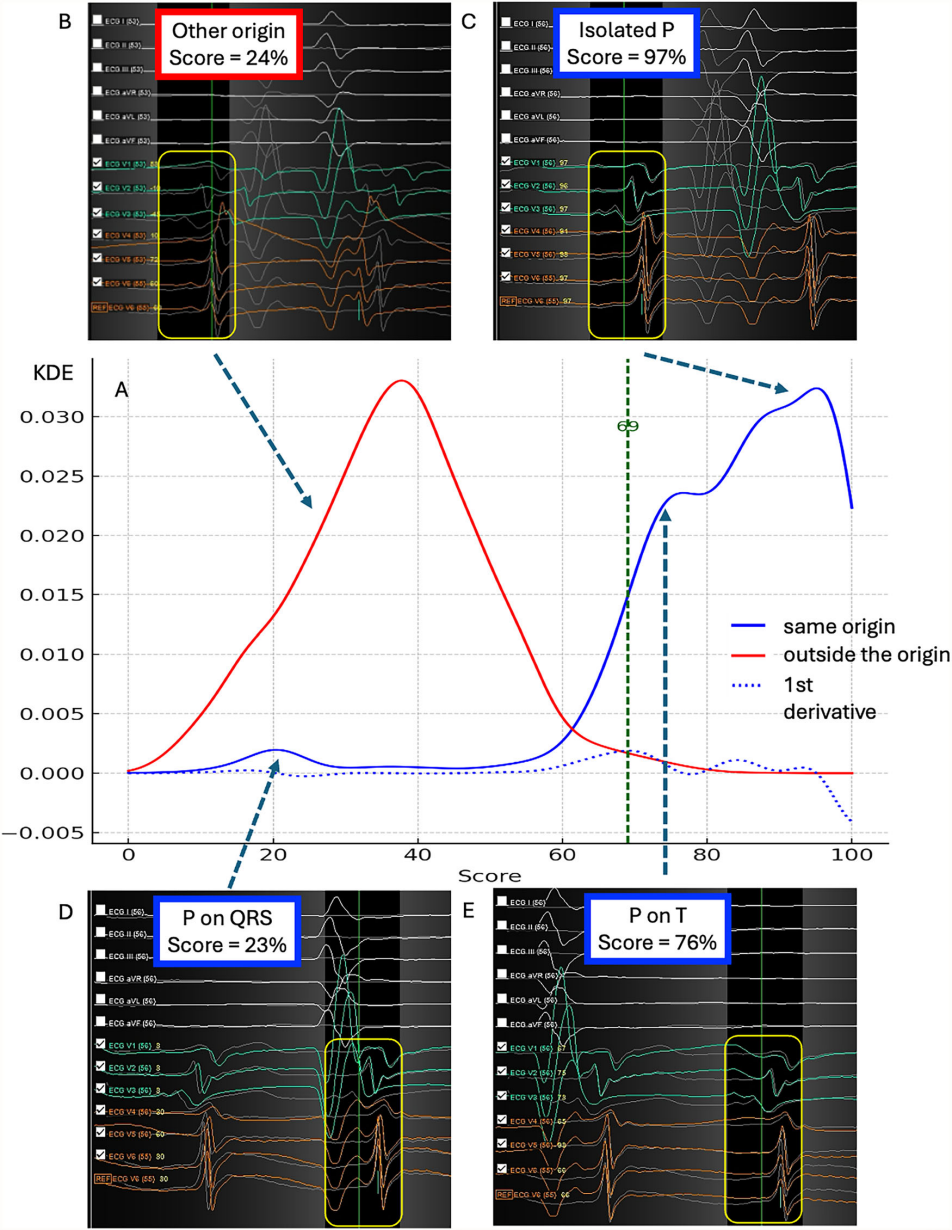
Definition of the IC pattern similarity score cutoff for beats of the same origin. (A) Dispersion of the IC pattern similarity score for the beats of the same origin (blue graph), its derivative (blue dotted graph) and for the beats outside the template origin (red graph). Inflection point analysis showed the optimal score cutoff = 69%. (B) Example of the low matching score between the IC patterns of the template and the beat from different origin. (C) Example of the high matching score between the IC patterns of the template and the beat from the same origin. (D) Example of the low matching score between the IC patterns of the template and the beat from the same origin due to overlay of the P‐wave and QRS. (E) Example of the moderate matching score between the IC patterns of the template and the beat from the same origin due to overlay of the P‐wave and T‐wave.

The KDE curve of the IC pattern similarity distribution showed a non‐gaussian form with multiple local maximums corresponding to the overlay of the P‐wave with the different segments of the QRST (Figure 1, 3). The AT cycle length had a moderate positive correlation with the median score (*r* = 0.49, *p* = 0.045) and a strong negative correlation with score variability (*r* = −0.88, *p* < 0.0001, Supporting Information S1: Figure S2). That indicates a lower median IC pattern similarity score and higher variability in the rhythms with shorter cycle lengths.

The ROC analysis suggested the IC similarity cutoff of 63% for the same SOO (sensitivity 95%, specificity 98%). The inflexion point analysis using the peak detection in the KDE first derivative curve revealed an optimal IC pattern similarity cutoff of 69% (sensitivity 90.4%, specificity 99%, Supporting Information S1: Figure S3).

### 3.2 Phase 2. Reliability and Accuracy of APM Using aICPM in Stable Rhythms

In 15 patients, 45 score maps were generated with a median of 72 points per map (IQR 54; 95). In three patients the origin of sustained focal AT in the RA and in 2 in the Liwith a median CL 420 ms (IQR 410; 450). In the other 10 patients who underwent first‐do PVI, the SOO was set by pacing in four regions: LA posterior and anterior wall, RA posterior and lateral wall. APM idenfied all rhythm origins with high reliability. The median best IC pattern matching was 98% (IQR 96%; 99%) for paced SOO and 88% (IQR 84%, 94%) for sustained AT. The top 10% IC pattern similarity area was 0.2 cm^2^ (IRQ 0.1; 0.5 mm^2^) for paced SOO and 0.5 cm2 (IQR 0.5; 0.6) for sustained AT (Supporting Information S1: Table S2). The spatial resolution of the map was higher for the paced origins as compared to the clinical AT (4% per mm [IQR 5.6; 2.5] vs. 2.5% [IQR 4%; 1.82%]). The median distance from the SOO to the best IC pattern similarity point was 2 mm (IQR 1; 3) for paced SOO and 4 mm (IQR 3; 4) for sustained AT. The best IC matching point was within the top 10% area in all cases.

### 3.3 Phase 3. Feasibility of APM Using aICPM for Localizing Non‐PV Triggers

**Figure 4:**
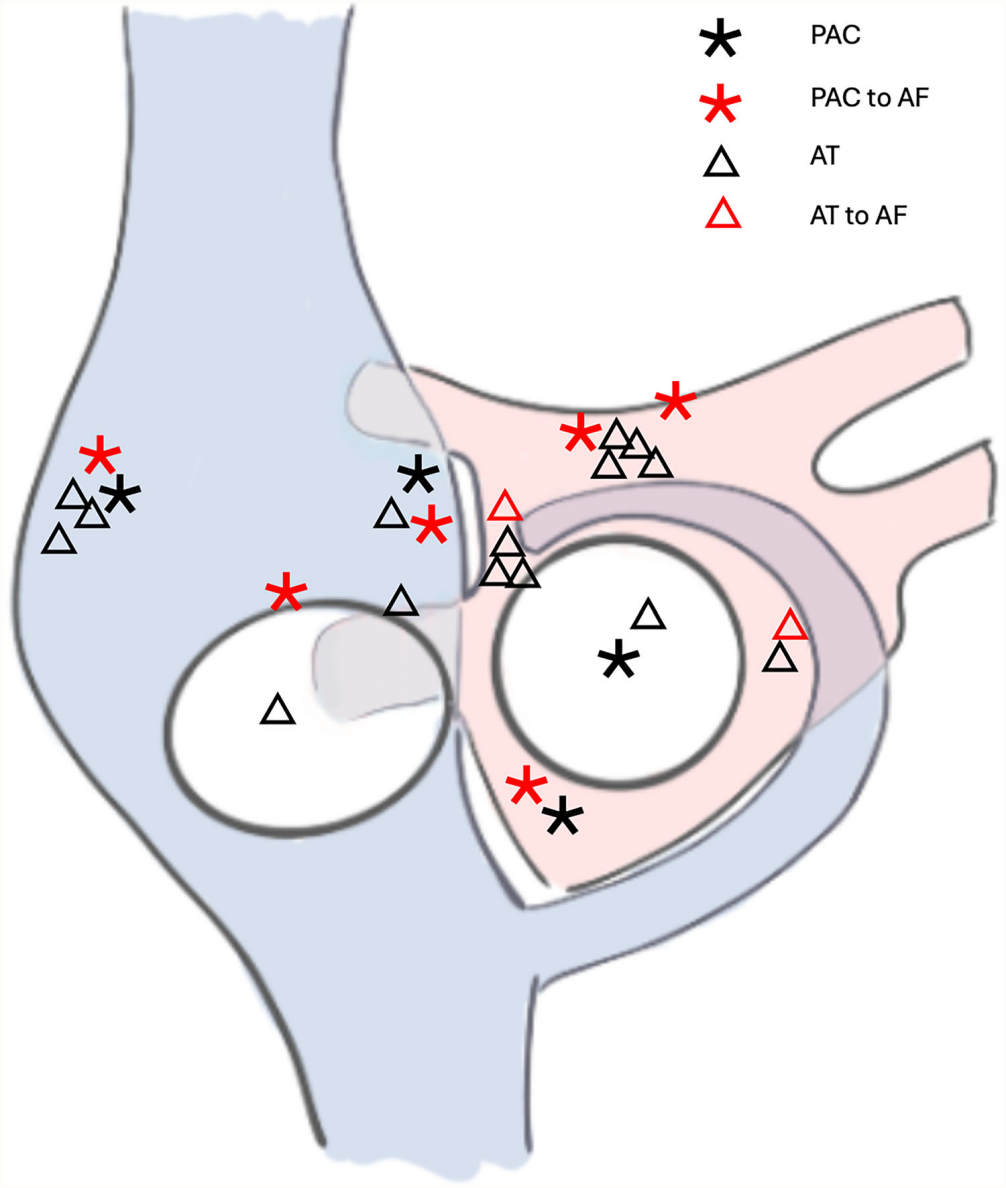
Anatomical locations of the non‐sustained atrial rhythm origins. AF, atrial fibrillation; AT, atrial tachycardia; PAC, premature atrial contraction.

A total of 45 out of 73 (61.6%) patients who underwent a redo ablation procedure for recurrence of AF and showed no low‐voltage substrate were included. In 6 out of 45 patients (13.3%), sustained AT or SVT were induced and ablated according to their mechanism. A reproducible induction of 27 non‐PV triggers was observed in 25 other (55,6%) patients (17 with nsAT and 10 with PAC, two patients exhibiting both). AF was initiated by a non‐PV trigger in 8 (29.6%) cases (Figure 4). The median age was 70.0 (IQR 62.5; 78.0), and eight patients (32%) were female. Seven patients (28%) had a persistent form of AF. The left atrial (LA) diameter was 43 (41; 45) mm, and the left ventricular ejection fraction was 55 (55; 60) %. The median cycle length/coupling interval of nsAT or PAC was 360 ms (300; 425) (Table 1). APM revealed the origin of nsAT or PAC in all 25 patients, with the most frequent location in the interatrial septum (*n* = 10, 37%), followed by the anterior wall of the LA (*n* = 6, 22.2%), the RA lateral wall (*n* = 5, 18.5%), LA mitral isthmus/vein of Marshall (*n* = 2, 7.4%), LA posterior wall (*n* = 2, 7.4%), RA posterior wall (*n* = 1, 3.7%) and anterior tricuspid valve (*n* = 1, 3.7%) (Figure 1, 3). The centrifugal distribution of the IC pattern similarity scores was observed in all cases with the median best IC pattern similarity score of 81% (IQR 75%; 89%), and the top 10% best IC pattern matching strata area of 0.6 cm² (IQR 0.2; 0.9). The spatial resolution of the map in the vicinity of the origin was 2.5% per mm (IQR 4; 1.82). The median time to perform APM and create a score map was 5.0 min (IQR 3.3; 6.3), with the median number of points per map of 106 (IQR 77; 155). Focal RF ablation successfully eliminated triggers in 23 of 25 patients. Two AF triggers from the vein of Marshall were eliminated by ethanol ablation. The median ablation time was 134 s (IQR 75;180), and the median ablation area was 2.0 cm² (IQR 1.2; 2.3). In all of cases, the areas of successful ablation were with within normal voltage, as patients with low‐voltage areas were excluded from the study. During a follow‐up period of 5.9 ± 1.87 months, 5 out of 25 patients (20%) experienced AF recurrence.

**Table 1:** Characteristic of the non‐PV triggers, parameters of their atrial pace mapping with automated intracardiac pattern matching.

#	Non‐PV trigger type		Cycle length/coupling interval (ms)	Non‐PV trigger location	Best IC pattern matching score (%)	Area of best 10% ICPM (cm²)	Number of points in score map	APM time (min)	RF ablation time (s)	RF ablation area (cm²)
1	PAC to AF		260	RA septal	62	0.2	55	17	99	2.6
2	AT		420	RA lateral	74	0.1	86	1.53	31	1.3
3	AT to AF		260	LA VOM	53	1.6	23	2.75	55	0.8
4	AT		280	RA lateral	80	1.6	154	1.7	248	3
5	AT		300	LA anterior	85	0.2	69	2.97	175	2
6	AT to AF		320	LA anterior	95	2.3	93	10.08	80	2.1
7	PAC to AF		400	RA lateral	69	3.1	78	6.22	88	1.5
8	AT		380	LA anterior	89	0.2	106	6.38	134	1.4
9	AT		240	LA VOM	98	0.6	76	3.2	306	5.1
10	AT		480	RA septal	89	0.2	80	4.35	203	0.45
11	AT		490	LA septal	75	0.2	72	4.03	184	2.8
12	PAC to AF		360	LA anterior	97	0.6	132	4.27	101	2.1
13	AT		430	RA lateral	76	0.3	151	5.67	173	2.3
14		PAC	440	LA posterior	82	0.7	123	3.42	222	8.1
15		PAC	410	RA septal	84	0.1	242	7.87	86	1.5
16		PAC to AF	280	LA septal	81	1.6	149	6.92	67	2.2
17		AT	400	LA septal	81	1	195	5.01	224	2.4
18		AT	700	RA lateral	77	0.4	92	5.07	174	2
19		AT	350	RA septal	71	0.2	115	3.9	30	1
20		AT	300	LA anterior	75	0.8	56	2.5	70	1
21		AT	310	LA anterior	92	0.8	92	3.9	144	0.8
22		PAC to AF	404	LA anterior	88	0.2	156	5.5	193	2
23		AT	310	RA posterior	52	0.8	220	8	28	0.7
24		AT	500	LA septal	92	0.1	65	2.1	120	1
25		PAC to AF	320	RA antrior	81	0.1	541	12	154	1.7
26		AT	220	LA posterior	82	1.8	208	6	148	2.1
27		PAC	520	LA septal	92	0.6	220	6	54	1.5

Abbreviations: AF, atrial fibrillation; APM, atrial pace mapping; AT, atrial tachycardia; IC, intracardiac; ICPM, intracardiac pattern matching; LA, left atrial; MI, mitral isthmus; PAC, premature atrial contraction; PV, pulmonary vein; RA, right atrial; RF, radiofrequency; VOM, vein of Marshall.

## 4 Discussion

What's new: This study demonstrates the feasibility of a novel approach of APM with aICPM using ECG pattern‐matching algorithms to localize non‐sustained atrial rhythms with high precision in a short time. The method offers a significant improvement in mapping efficiency and resolution compared to traditional techniques, enabling better management of challenging atrial arrhythmias including individualized ablation strategies for AF.

Automated IC pattern matching for atrial pace mapping. Due to the oftentimes poor reproducible inducibility of non‐PV triggers, conventional techniques using activation mapping have clear limitations. Identifying the location of non‐PV triggers, if possible, is often imprecise and can be associated with long procedure duration. The proposed strategy, using unipolar pattern and automatic pattern matching allowed precise identification (top 10% best IC pattern matching area was 0.6 cm²) within a few minutes (median time 5.0 min).

APM is a helpful mapping technique in the cases of non‐stable atrial rhythms like non‐sustained or bumped AT or non‐PV triggers where activation mapping is not applicable [9]. The automatic electrogram matching algorithms recently introduced in the mapping systems may enhance APM, which hereto relied on the subjective analysis of the P‐wave morphology and local timings of IC signals of the origin beat. The workflow for the APM with aICPM utilizing the dedicated algorithm of the Carto‐3 system was previously described by Yamashita et al. [10, 11, 12]. This Carto‐3 algorithm was developed to track the stability of atrial rhythm and can be helpful when applied during APM. However, there is no direct way to represent the distribution of the matching score in the 3D map. A workaround with the creation of pseudo‐activation maps by assigning the matching score values to the local activation time values is time‐consuming. The authors used an averaged matching score of several beats manually assigned to the point, introducing some degree of subjectivity. That was probably the main reason for the low density of the IC pattern match maps reported by Yamashita et al., which had only 9 to 16 points per map.

Integration of the unipolar IC signals into automatic ECG matching algorithm. The EnsiteX system lacks aICPM algorithm but has a pattern‐matching module for the surface ECG that automatically creates the “score” map, showing pattern similarity distribution on the 3D surface. Users can select specific ECG leads for matching. In our study, six referent electrodes were connected to precordial leads, and their unipolar IC signals were processed by the ECG matching algorithm. Using only precordial leads enabled aICPM with six IC signals and straightforward score map generation. The main advantage of our novel approach is the automatic acquisition of the points into the score map instead of manual acquisition and reannotation reported by Yamashita et al. This approach yielded a much higher map density (106 vs. 12 points per map) in comparable time (5.0 vs. 4.1 min).

Reproducibility of the IC pattern from common origin. In our study, we targeted only reproducible ectopy from the same SOO based on IC pattern morphology. In Phase 1, we established an IC pattern similarity cutoff to confirm ectopic beats from the same SOO by comparing the distributions of the matching scores of beats from the same versus different SOOs. IC pattern dispersion was attributed to P‐wave overlay with QRS and ST waves, respiratory cycles, noise, and minor electrode shifts. A 69% similarity score accurately predicted the same SOO with 99% specificity and 95% sensitivity. The negative correlation between cycle length and IC pattern dispersion likely reflects inter‐atrial conduction variability resulting in the fluctuation of the timing between referent catheters. allowing thresholds to be adjusted for slower versus faster rhythms. Therefore, thresholds can be adjusted for slower vs. faster rhythms.

Validation and feasibility of the novel technique. Before applying to unstable non‐PV triggers, in Phase 2, we validated the technique on sustained focal AT and paced rhythms of known origin. APM with aICPM identified the SOO with high precision, with median IC similarity scores significantly higher in Phase 2 than for non‐PV triggers in Phase 3 (98% vs. 81%). That can be explained by slower‐paced rhythms (CL 500 ms) used in 40 out of 45 maps in Phase 2. For the same reason, the score maps for slower rhythms also inherited a higher spatial resolution of 4% per mm compared to 2.5% per mm for faster rhythms. In Phase 3, this novel approach successfully localized ectopy origins in non‐sustained rhythms. Automatic score maps with more than 100 points, created in just 5 min, were denser than manual methods with the Carto system.

Robustness of the algorithm and spatial resolution. The similarity score, based on correlation, emphasizes temporal alignment of voltage curves within a specified time window, making the absolute voltage values less critical. This approach is advantageous when the absolute amplitudes or signal shapes vary due to contact quality while the activation timing relative to a reference remains consistent. In essence, the algorithm prioritizes penalizing temporal misalignments over amplitude discrepancies, making it less sensitive to fluctuations in the absolute voltage of the waveforms. For instance, as illustrated in Figure 2, the template and the paced beat morphologies are not identical despite the referent electrodes remaining in the same location. However, the overall similarity score remains high at 81%, as the potentials align closely in time. This underscores the algorithm's strength in evaluating waveform similarity based on timing rather than amplitude discrepancies. Notably, pacing artifacts were typically outside the matching window or had minimal influence on IC signal morphology (Figure 2).

An equal number of referent electrodes placed in the RA and LA might be essential to maintain the balance of weights in the matching algorithms. Yamashita et al. used 4 unipolar signals in the right and four in the left atrium. The Carto‐3 aICPM algorithm utilizes the dV/dt of these unipolar signals to weight the signals in the analysis to calculate the total matching score [12]. The EnsiteX surface ECG matching algorithm computes the total similarity score by averaging the matching scores of the leads included in the analysis. Our approach, using three unipolar signals in each atrium (limited by precordial ECG leads), still achieved high spatial resolution and accuracy. The top 10% IC pattern similarity area was just 0.6 cm². In other words, when moving the pacing site 4 mm away from the site with the best IC pattern similarity, the score drops by 10%.

This study demonstrates the feasibility of APM with aICPM for localizing non‐sustained rhythms, that would have been unmappable with traditional activation mapping. The automatic matching algorithm introduces objectivity and reproducibility to the mapping process. Multicenter randomized trials with larger populations are needed to confirm its superiority over traditional non‐PV trigger mapping and assess long‐term clinical benefits for AF patients.

## 5 Limitations

This study has several limitations. The small patient cohort and short follow‐up limit conclusions on the clinical benefit of non‐PV trigger ablation. Some patients lacked true AF triggers, and it remains unclear if recurrence stemmed from inadequate mapping or ablation. This pilot study primarily assessed intraprocedural feasibility. Electrical wavefront conduction along the Bachmann bundle, CS, and crista terminalis may influence IC pattern areas. Matching septal origins depends on pacing output; low output was used to avoid biatrial capture. The epicardial VOM poses challenges for pace mapping, as the guide catheter replaces the CS catheter for VOM cannulation. High‐output endocardial pacing was used in these cases. Isoproterenol for arrhythmia induction may affect reference waveforms, and score fluctuations due to respiration highlight the need for breath‐phase filtering in the algorithm to ensure data consistency.

## 6 Conclusion

This novel approach to atrial pace mapping leverages the integration of intracardiac signals with the ECG pattern‐matching algorithm in the mapping system. It enables precise localization of previously unmappable, non‐sustained atrial rhythms by generating detailed pace maps automatically and efficiently within a short mapping time. Future randomized clinical trials with larger patient cohorts are essential to validate the clinical utility and long‐term benefits of this approach.

**Video 1:** Embedded video. Video content can be viewed at https://onlinelibrary.wiley.com/doi/10.1111/jce.16734

## 7 Clinical Perspectives

This novel atrial pace mapping approach integrates intracardiac signals with an ECG pattern‐matching algorithm to localize ectopic foci in non‐sustained atrial rhythms. The automated method enhances reliability and objectivity, offering faster mapping and higher‐resolution maps compared to traditional techniques. The approach is particularly useful for identifying and ablating arrhythmogenic foci in challenging cases, such as non‐PV triggers and atrial tachycardias, improving outcomes in previously unmappable rhythms. It utilizes existing mapping systems with minimal additional training, making it feasible for routine clinical practice.

Further technical advancements, like direct integration of the multiple unipolar signals into the IC pattern algorithm without need for passing the signals to ECG, incorporating breath‐phase filtering and refined algorithms, tracking for atrial capture, could enhance its precision and applicability to complex cases. While promising, larger randomized trials are needed to confirm its clinical benefits and long‐term impact. If validated, this method could significantly advance personalized ablation management in patients with atrial fibrillation.

## Conflicts of Interest

The authors declare no conflicts of interest.

## Supporting information

Supplemental Material.

## Data Availability

The data that support the findings of this study are available from the corresponding author upon reasonable request.

## References

[1] W. Ju , B. Yang , H. Chen , et al., “Mapping of Focal Atrial Tachycardia With an Uninterpretable Activation Map After Extensive Atrial Ablation: Tricks and Tips,” Circulation: Arrhythmia and Electrophysiology 7, no. 4 (August 2014): 598–604. 25017400 10.1161/CIRCEP.114.001508

[2] E. Lian , S. Willert , R. Krüger , T. Demming , D. Frank , and V. Maslova , “Atrial Pace Mapping Using Automatic Intracardiac Pattern Matching for Ablation of Non‐Sustained Atrial Tachycardia: A Case Report,” HeartRhythm Case Reports 10, no. 11 (2024): 794–797, 10.1016/J.HRCR.2024.07.022. 39664663 PMC11628854

[3] S. Higa , L. W. Lo , and S. A. Chen , “Catheter Ablation of Paroxysmal Atrial Fibrillation Originating From Non‐Pulmonary Vein Areas,” Arrhythmia & Electrophysiology Review 7, no. 4 (2018): 1, 10.15420/AER.2018.50.3. PMC6304799 30588316

[4] P. Santangeli , E. S. Zado , M. D. Hutchinson , et al., “Prevalence and Distribution of Focal Triggers in Persistent and Long‐Standing Persistent Atrial Fibrillation,” Heart Rhythm 13, no. 2 (2016): 374–382, 10.1016/J.HRTHM.2015.10.023. 26477712

[5] D. G. Della Rocca , L. Di Biase , S. Mohanty , et al., “Targeting Non‐Pulmonary Vein Triggers in Persistent Atrial Fibrillation: Results From a Prospective, Multicentre, Observational Registry,” EP Europace 23, no. 12 (2021): 1939–1949, 10.1093/EUROPACE/EUAB161. 34417816

[6] M. Thind , M. R. Arceluz , I. Lucena‐Padros , et al., “Identifying Origin of Nonpulmonary Vein Triggers Using 2 Stationary Linear Decapolar Catheters,” JACC: Clinical Electrophysiology 9, no. 11 (2023): 2275–2287, 10.1016/J.JACEP.2023.07.017. 37737775

[7] K. C. Man , K. K. Chan , P. Kovack , et al., “Spatial Resolution of Atrial Pace Mapping as Determined by Unipolar Atrial Pacing at Adjacent Sites,” Circulation 94, no. 6 (1996): 1357–1363, 10.1161/01.CIR.94.6.1357. 8822993

[8] P. M. Kistler , D. Chieng , I. R. Tonchev , H. Sugumar , et al., “P‐Wave Morphology in Focal Atrial Tachycardia: An Updated Algorithm to Predict Site of Origin,” JACC Clin Electrophysiol 7, no. 12 (December 2021): 1547–1556. 34217661 10.1016/j.jacep.2021.05.005

[9] K. Hayashi , S. Mathew , C. H. Heeger , et al., “Pace Mapping for the Identification of Focal Atrial Tachycardia Origin,” Circulation: Arrhythmia and Electrophysiology 9, no. 7 (2016): e003930, 10.1161/CIRCEP.116.003930. 27390210

[10] K. Yamashita , K. Furuya , Y. Sato , et al., “Intracardiac Electrogram–Based Atrial Pace Mapping for Detecting the Earliest Activation Site in Atrial Arrhythmias,” Heart Rhythm 21, no. 8 (February 2024): 1400–1408, 10.1016/J.HRTHM.2024.02.028. 38369035

[11] K. Furuya , D. Kumazawa , Y. Mizuno , K. Onodera , T. Nomura , and K. Yamashita , “A Novel Dual‐Chamber Reference Technique to Detect Premature Atrial Complexes With Non–Pulmonary Vein Foci,” HeartRhythm Case Reports 9, no. 5 (2023): 287–290, 10.1016/J.HRCR.2023.01.014. 37324966 PMC10265126

[12] K. Yamashita , K. Furuya , D. Kumazawa , Y. Mizuno , K. Onodera , and T. Nomura , “Novel Atrial Pace‐Mapping Technique Based on Dual‐Chamber Electrograms to Detect Non–Pulmonary Vein Foci,” HeartRhythm Case Reports 9, no. 10 (2023): 723–727, 10.1016/J.HRCR.2023.07.014. 38047201 PMC10691955

