# Supplementary material for: A Novel Approach for Localizing Non‐Sustained Atrial Arrhythmias: Atrial Pace‐Mapping With Automatic Intracardiac Pattern Matching
Source: J Cardiovasc Electrophysiol. 2025 Jun 3;36(8):1798–807. doi: 10.1111/jce.16734 (PMC13020650; doi:10.1111/jce.16734)
Supplement: Supplementary file 2 — Supplemental Material. [file JCE-36-1798-s001.docx]

Supplemental Material


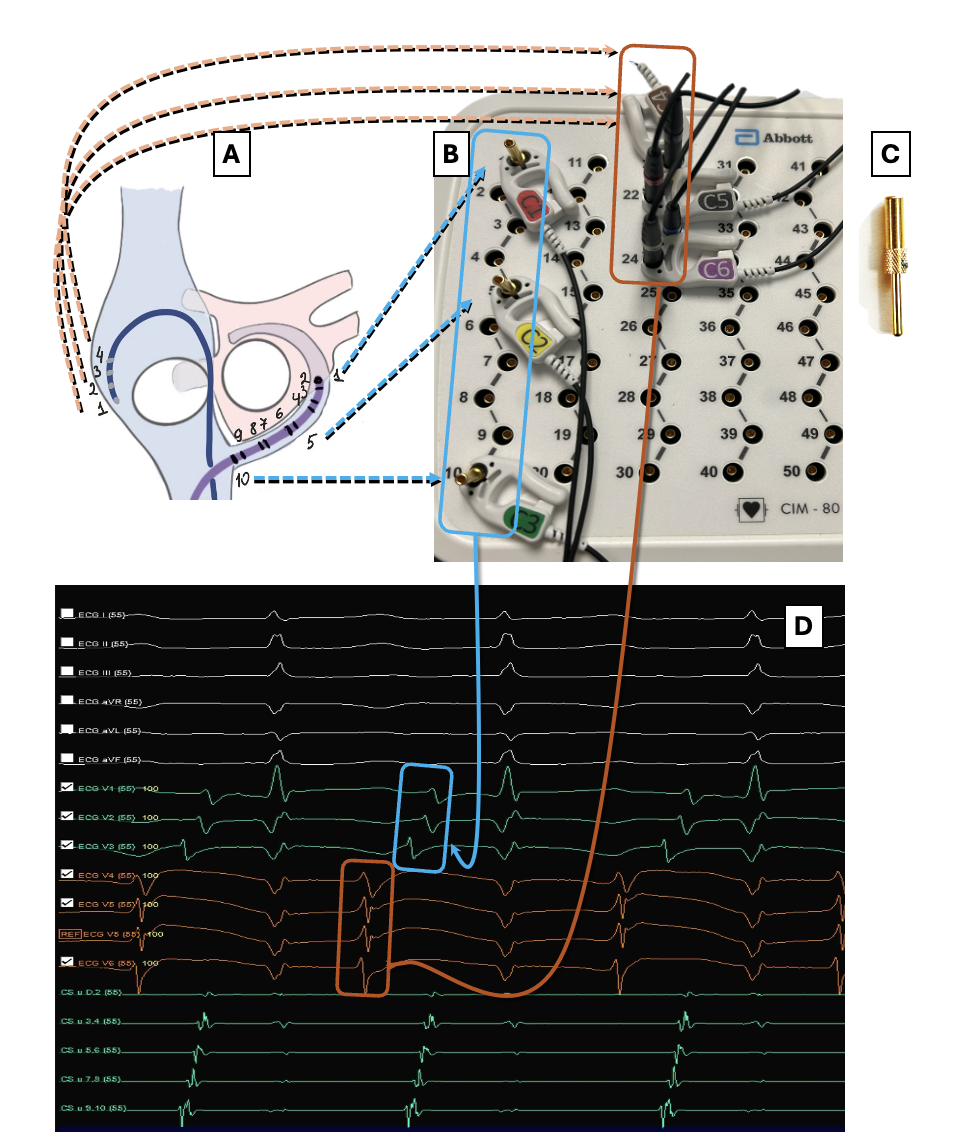


Supplemental Figure 1. The technical setup for atrial pace mapping with double chamber reference. (A) Schematic illustration of reference catheters positioned in the coronary sinus and the right atrium; (B) Connection of the precordial leads to the three poles of the coronary sinus catheter and three poles of the right atrial catheter. Note that the cable pins of the decapolar catheter are detached to illustrate that the connection of the ECG leads to pin extensions. (C) The custom-made adapter provides an extension of the cable pins. (D) Six intracardiac unipolar electrograms were recorded from the coronary sinus and right atrium and passed to the V1-V6 ECG leads.


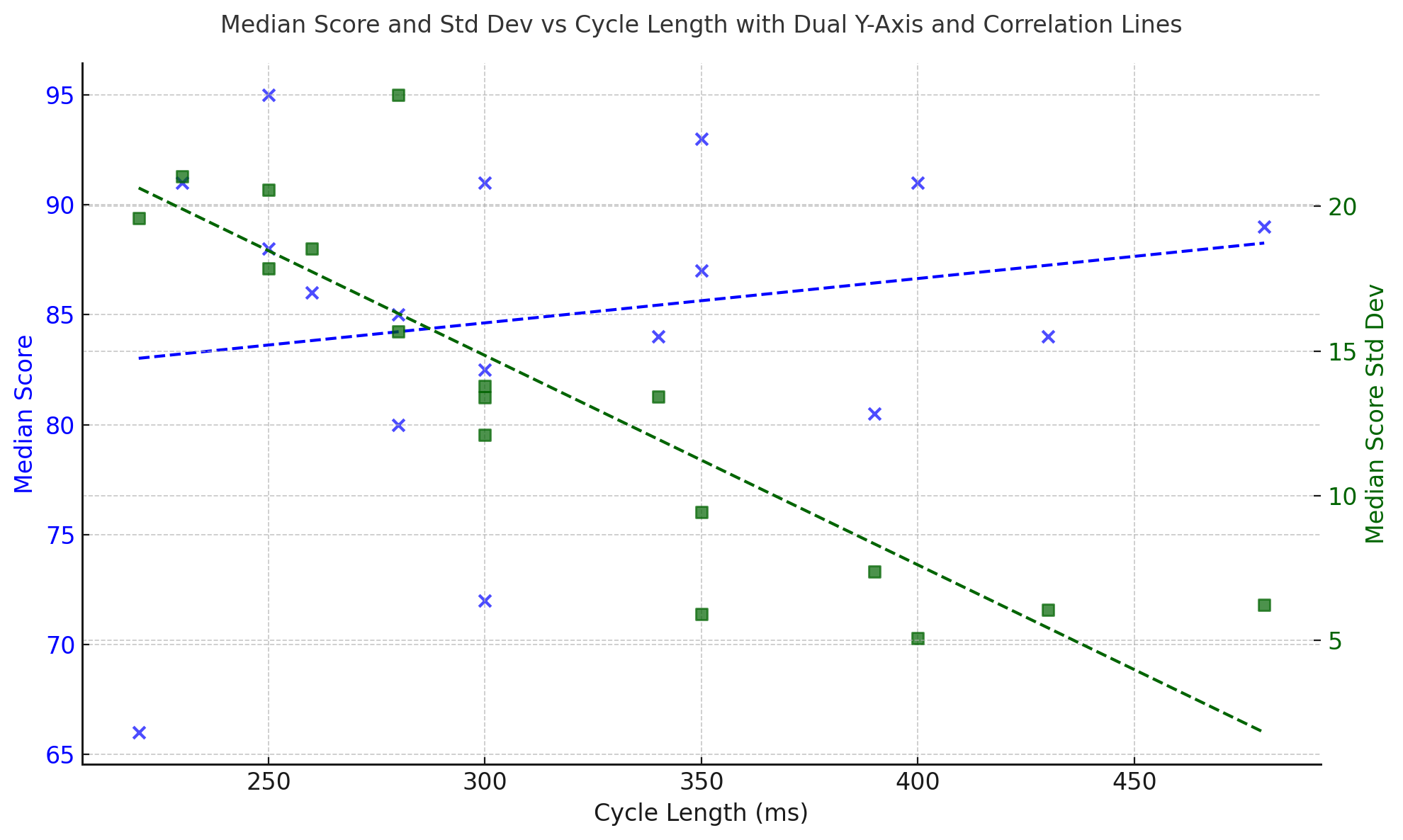


Supplement Figure 2. The scatter plot shows a positive correlation between cycle length and the IC pattern similarity score and a strong negative correlation between cycle length and standard deviation of similarity score.


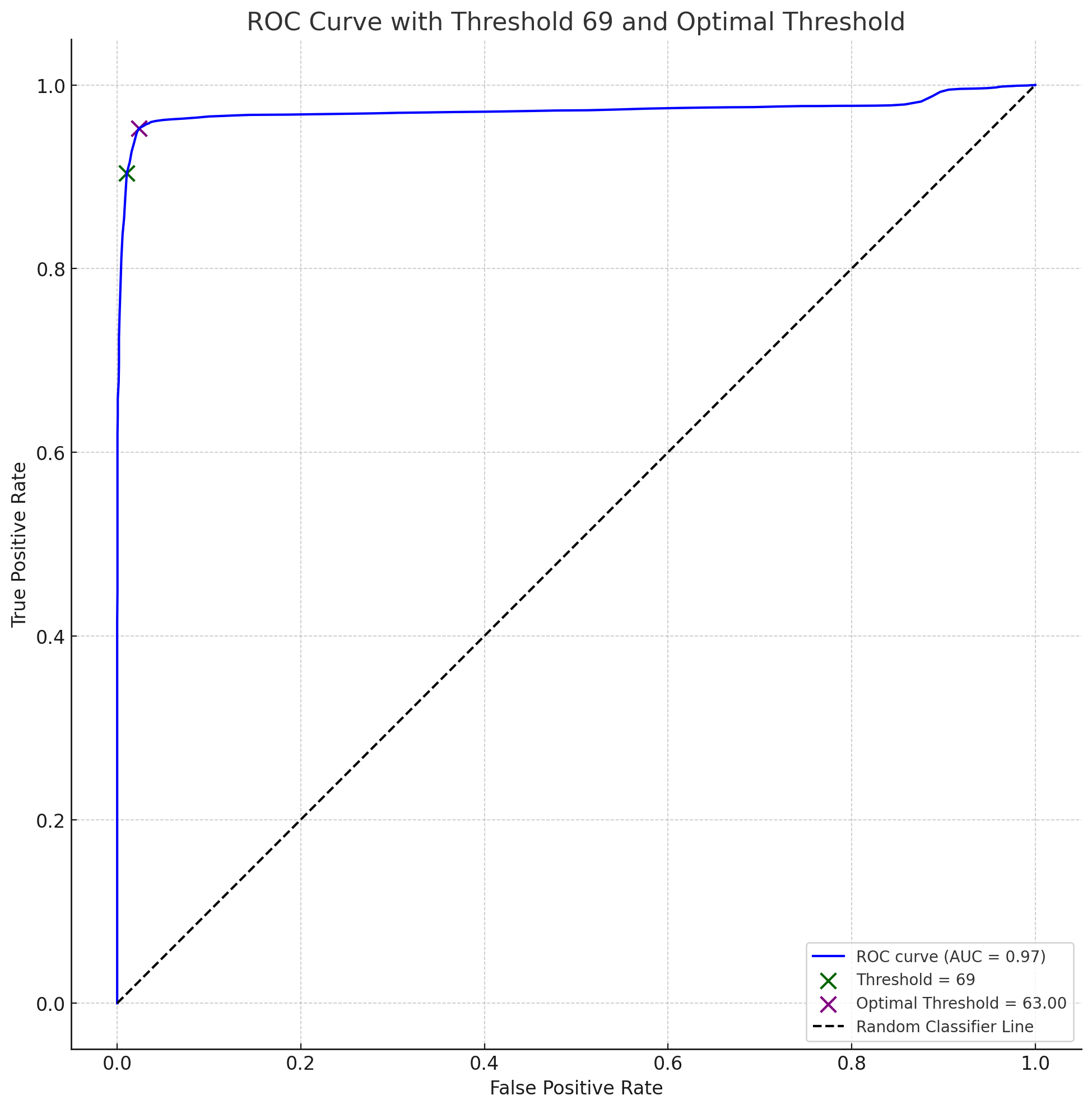


Supplement Figure 3. ROC analysis shows the optimal cutoff value of 63% for the IC pattern similarity score of the same origin. AUC, area under the curve, ROC, receive operator curve, IC, intracardiac. Note the cutoff value of 69% defined by inflexion point analysis of the score distribution inheriting lower sensitivity but higher specificity.

Supplemental Video. Atrial pace mapping with automatic intracardiac pattern matching using double chamber reference. Three poles of the coronary sinus catheter and three of the high right atrium (HRA) catheter are connected to the precordial lead. The intracardiac pattern of non-sustained AT is captured and set as the template. The triggering is set to the V4 (HRA distal unipolar) signal. The score map of the left atrium is automatically created while pacing from the ablation catheter with contact force monitoring.

Supplement Table 1. Cycle length of sustained atrial tachycardia, median IC pattern similarity score, and standard deviation.

| **Cycle Length (ms)** | **Median Score** | **Median Score Std Dev** |
| --- | --- | --- |
| 220 | 66.0 | 19.60 |
| 230 | 91.0 | 21.02 |
| 250 | 95.0 | 20.56 |
| 250 | 88.0 | 17.85 |
| 260 | 86.0 | 18.54 |
| 280 | 85.0 | 23.84 |
| 280 | 80.0 | 15.68 |
| 300 | 82.5 | 13.79 |
| 300 | 91.0 | 12.09 |
| 300 | 72.0 | 13.41 |
| 340 | 84.0 | 13.43 |
| 350 | 87.0 | 9.44 |
| 350 | 93.0 | 5.91 |
| 390 | 80.5 | 7.38 |
| 400 | 91.0 | 5.10 |
| 430 | 84.0 | 6.06 |
| 480 | 89.0 | 6.25 |

Supplement Table 2. Accuracy metrics for APM with aICPM for rhythms of known origin in 10 patients with paced rhythms from 4 locations and 5 patients with sustained focal atrial tachycardia. CL, cycle length; IC, intracardiac; SOO, site of origin

| **Patient #** | **Origin pacing site** | **CL** | **Number of points** | **Best IC pattern matching** | **Area of the best 10% IC pattern matching** | **Distance best score to SOO, mm** |
| --- | --- | --- | --- | --- | --- | --- |
| Paced 1 | LA post | 500 | 68 | 99% | 0.05 | 2 |
|  | LA ant | 500 | 68 | 99% | 0.1 | 1 |
|  | RA post | 500 | 115 | 98% | 0.8 | 4 |
|  | RA lat | 500 | 95 | 99% | 0.7 | 1 |
| Paced 2 | LA post | 500 | 28 | 98% | 1.5 | 4 |
|  | LA ant | 500 | 42 | 94% | 0.7 | 2 |
|  | RA post | 500 | 58 | 98% | 0.1 | 2 |
|  | RA lat | 500 | 88 | 98% | 0.2 | 2 |
| Paced 3 | LA post | 500 | 110 | 93% | 1.2 | 1 |
|  | LA ant | 500 | 128 | 99% | 0.1 | 2 |
|  | RA post | 500 | 87 | 100% | 0.2 | 1 |
|  | RA lat | 500 | 109 | 89% | 0.1 | 3 |
| Paced 4 | LA post | 500 | 101 | 98% | 0.05 | 3 |
|  | LA ant | 500 | 107 | 99% | 0.05 | 1 |
|  | RA post | 500 | 108 | 97% | 0.3 | 2 |
|  | RA lat | 500 | 111 | 100% | 0.1 | 1 |
| Paced 5 | LA post | 500 | 95 | 99% | 0.1 | 4 |
|  | LA ant | 500 | 71 | 99% | 0.05 | 2 |
|  | RA post | 500 | 74 | 96% | 0.5 | 1 |
|  | RA lat | 500 | 47 | 98% | 0.2 | 2 |
| Paced 6 | LA post | 500 | 109 | 100% | 0.5 | 3 |
|  | LA ant | 500 | 94 | 99% | 0.6 | 1 |
|  | RA post | 500 | 70 | 99% | 0.5 | 2 |
|  | RA lat | 500 | 55 | 99% | 0.3 | 1 |
| Paced 7 | LA post | 500 | 62 | 98% | 0.4 | 0 |
|  | LA ant | 500 | 42 | 98% | 0.5 | 1 |
|  | RA post | 500 | 22 | 91% | 0.1 | 2 |
|  | RA lat | 500 | 21 | 99% | 0.1 | 2 |
| Paced 8 | LA post | 500 | 54 | 96% | 0.1 | 1 |
|  | LA ant | 500 | 90 | 87% | 0.05 | 3 |
|  | RA post | 500 | 113 | 98% | 0.6 | 1 |
|  | RA lat | 500 | 81 | 83% | 0.2 | 1 |
| Paced 9 | LA post | 500 | 68 | 99% | 2 | 1 |
|  | LA ant | 500 | 55 | 99% | 0.5 | 2 |
|  | RA post | 500 | 73 | 99% | 0.2 | 4 |
|  | RA lat | 500 | 46 | 97% | 0.4 | 1 |
| Paced 10 | LA post | 500 | 75 | 98% | 0.2 | 1 |
|  | LA ant | 500 | 49 | 91% | 0.05 | 3 |
|  | RA post | 500 | 43 | 99% | 0.1 | 2 |
|  | RA lat | 500 | 49 | 97% | 0.05 | 2 |
| sustained FAT 1 | LA anteroseptal | 410 | 64 | 84% | 0.6 | 4 |
| sustained FAT 2 | RA crista terminalis | 420 | 110 | 94% | 0.5 | 4 |
| sustained FAT 3 | LA septal | 400 | 43 | 88% | 0.3 | 3 |
| sustained FAT 4 | RA tricuspid valve | 450 | 89 | 75% | 0.5 | 3 |
| sustained FAT 5 | RA crista terminalis CL | 510 | 72 | 98% | 0.7 | 5 |
